# An elastin-like recombinamer-based bioactive hydrogel embedded with mesenchymal stromal cells as an injectable scaffold for osteochondral repair

**DOI:** 10.1093/rb/rbz023

**Published:** 2019-05-20

**Authors:** Filippo Cipriani, Blanca Ariño Palao, Israel Gonzalez de Torre, Aurelio Vega Castrillo, Héctor José Aguado Hernández, Matilde Alonso Rodrigo, Angel José Àlvarez Barcia, Ana Sanchez, Verónica García Diaz, Monica Lopez Peña, José Carlos Rodriguez-Cabello

**Affiliations:** 1 Technical Proteins Nanobiotechnology S.L., Paseo Belén 9A, Valladolid 47011, Spain; 2 Departamento de traumatología, Hospital Clínico de Valladolid, Av. Ramón y Cajal 3, Valladolid 47003, Spain; 3 Bioforge, University of Valladolid CIBER-BBN, Paseo de Belén 19, Valladolid 47011, Spain; 4 SIBA—UVA: servicio investigación y bienestar animal, University of Valladolid, C/Plaza de Santa Cruz 8, Valladolid 47002, Spain; 5 Instituto de Biología y Genética Molecular (IBGM), Universidad de Valladolid y CSIC, Calle Sanz y Fores 3, Valladolid 47003, Spain; 6 Facultad de veterinaria, Campus Universitario, Avda. Carballo Calero s/n, Lugo 27002, Spain

**Keywords:** biopolymer, animal test, cartilage, bone

## Abstract

The aim of this study was to evaluate injectable, *in situ* cross-linkable elastin-like recombinamers (ELRs) for osteochondral repair. Both the ELR-based hydrogel alone and the ELR-based hydrogel embedded with rabbit mesenchymal stromal cells (rMSCs) were tested for the regeneration of critical subchondral defects in 10 New Zealand rabbits. Thus, cylindrical osteochondral defects were filled with an aqueous solution of ELRs and the animals sacrificed at 4 months for histological and gross evaluation of features of biomaterial performance, including integration, cellular infiltration, surrounding matrix quality and the new matrix in the defects. Although both approaches helped cartilage regeneration, the results suggest that the specific composition of the rMSC-containing hydrogel permitted adequate bone regeneration, whereas the ELR-based hydrogel alone led to an excellent regeneration of hyaline cartilage. In conclusion, the ELR cross-linker solution can be easily delivered and forms a stable well-integrated hydrogel that supports infiltration and *de novo* matrix synthesis.

## Introduction

Chondral and osteochondral defects in the articular cartilage of the knee and in other joints caused by traumatic and non-traumatic injuries tend to progress to degenerative osteoarthritis over time. This scenario usually leads to total replacement of the joint with prosthesis [1]. Several types of cartilage are known, including the articular hyaline cartilage, which is a smooth, pearly bluish layer with a width of 2–4 mm that covers the articular surfaces [2]. Articular hyaline cartilage is a highly specialized tissue characterized by its unique mechanical properties [2]; it has a structural role adsorbing the pressure overload the cartilage, and a functional role allowing the friction-less sliding of the articular surface [3, 4]. When the cartilage layer is damaged, the structural components of hyaline cartilage (proteoglycans and glycosaminoglycans) tend to leak from it, reducing the ability to absorb the pressure overload [5]. Consequently, the functional capacity of friction-less sliding decreases, indeed, due to the remodeling of the layer, the water diffusion into the cartilage is reduced. Hyaline cartilage diseases bring synovitis, which progress to the inflammation of the articular layer [6]. In mammals, the ability of articular cartilage to durably repair decreases soon after birth and is almost completely lost by early adulthood [7]. Generally, the regenerated cartilage is rich in type I collagen (fibrocartilage) instead of containing type II collagen. Collagen type II provides tensile ability to the cartilaginous matrix and is essential for articular hyaline functional capacities [8], whereas fibrocartilage is unable to maintain the biomechanical characteristics of articular cartilage [2, 4, 5]. All treatments currently used to restore the hyaline articular surface are unsatisfactory [2], although several alternatives have been probed to promote the regeneration of damaged cartilage. In particular, the development of novel tissue-engineering methods has started to play an important role [9, 10]. Mesenchymal stromal cell (MSC) therapy [11–13] is a method that utilizes pluripotent cells, which can differentiate into various cell types, such as chondrocytes and osteocytes. As a result, these cells are good candidates for the treatment of musculoskeletal lesions [14, 15]. MSCs are available from different auto-, allo- and xenogeneic sources [16]. The first two options offer an immunologically safer approach, whereas the latter hugely increases the availability of MSCs [17]. Although there are several studies with successful results using xenogeneic MSCs in different animal hosts [18], only autologous [19] or allogenic cells [13] have been successfully used in humans, with negligible immunological response [20]. Moreover, in the case of osteochondral application, it must be taken into account that articular cartilage is considered an immunoprivileged tissue, indeed, due to its avascularity, the immune system has some limitations for the detection of implanted tissue [21]. The suspension of MSCs in a scaffold as a cell-carrier enhances the persistence of the implanted cells at the treatment site [2]. Taking into account that the majority of wet articular cartilage is formed by water [22], hydrogels represent one of the most promising solutions for cartilage repair applications. Moreover, it is important to consider that, during surgery, it is crucial to minimize the severity of the intervention [23]. One advantage of the arthroscopic technique is that it can reduce infection risk and recovery time compared to open joint surgery. In light of this, the use of injectable hydrogels is of special interest because they are compatible with arthroscopic methods [23].

The use of recombinant DNA techniques has brought new materials to the biomedical field, discovering new matrices for tissue engineering (TE) applications. An important role is played by elastin-like recombinamers (ELRs); they are based on the repetitive pentapeptide sequence Val−Pro−Gly−X−Gly (VPGXG)_*n*_, where the guest residue (X) is any amino acid except l-proline [24]. The thermosensitivity shown by ELRs is defined by the transition temperature (*T*_t_). It depends on the charge of protein conformations and on the polarity of the amino acids that composed the ELRs [25, 26]. Moreover, a great advantage of the ELRs is the ability to form different structures, among which a hydrogel is one of the most common for regenerative medicine application [9]. As pointed out above, the ELRs show thermosensitivity, thus meaning that hydrogels, which are stable at body temperature, can be formed whenever the *T*_t_ of the ELR is lower than this temperature [27]. Several studies have shown how different types of ELRs can be used in some of the most challenging fields of tissue regeneration, such as cardiovascular [28], ocular prosthesis [29] and osteochondral applications [30, 31], among others [27].

The incorporation of cells into biomaterials can help to overcome some limitations of using cells or biomaterials alone. For instance, an ELR-based hydrogel can serve as a scaffold to allow MSCs to orchestrate tissue regeneration. Moreover, considering the extraordinary compatibility of ELRs, the 3D hydrogel structure can mimic the properties of the extracellular matrix (ECM), thereby supporting the regeneration process.

In this study, in order to promote cell attachment and stimulate matrix production, we developed an appropriate ELR-based bioactive hydrogel composition that provides an adequate balance of properties, such as mechanical support [32], to foster cell adhesion and proliferation. Given their recombinant nature, ELRs were designed to contain bioactive sequences, such as the extensively studied RGD sequence, which supports cell adhesion via integrins [33], CS5 human fibronectin REDV for efficient cell attachment [28, 34] and VGVAPG as an elastase target domain (human leukocyte elastase I) to provide increased proteolytic sensitivity and increased functionality to the scaffold [35, 36]. In this study, we obtained a homogeneous embedding of rabbit MSCs (rMSCs) in the ELR solution at a temperature below body temperature, and injected this composition as a cell-scaffold system for osteochondral repair. This ELR-based bioactive hydrogel exhibited a cell-friendly environment, thus improving cartilage regeneration both with and without rMSCs embedded.

## Materials and methods

### Ethical approval

All procedures regarding the collection of rMSCs specified below were approved by the Ethics Committee of the University Hospital of Valladolid (Spain) in accordance with the Declaration of Helsinki (1975), as revised in 2013. All animal experiments were conducted in accordance with the institutional guidelines for the care and use of experimental animals of the University of Valladolid (Spain) in accordance with Directive 2010/63/EU (Resolution Number 2010/2/23).

### Rabbit mesenchymal stem cell collection

Bone marrow was extracted from the tibias and fibulas of white New Zealand male rabbits and collected in sterile tubes (Falcon^®^ A Corning Brand, Ref. 352070) previously damped with a heparinized saline solution of Phosphate Buffered Saline (PBS, Gibco Ref. 20012-068) and 5% Heparin Sodium (Chiesi Spain S.A.U) to avoid coagulation. Bone marrow samples were kept at 4°C until they were processed within 24 h. A fraction of mononuclear cells (MNCs) was selected using a density gradient method with Ficoll-Paque PREMIUM (GE Healthcare Ref. 17-5442-02). At the end of this process, counting and viability controls were performed using the Trypan Blue exclusion method with a Neubauer Chamber. After the selection process, MNCs were seeded at a density of 190 × 10^3^ cells/cm^2^ and kept in culture at 37°C and 10% CO_2_ with Dulbecco’s modified Eagle medium (DMEM) 4.5 g/l d-glucose (Gibco, Ref. 31966-021) supplemented with 0.041 mg/ml of gentamicin (Gibco, Ref. 15710-049) and 20% Fetal Bovine Serum (FBS, Gibco). Every 3 or 4 days, the appearance of the cell monolayer was observed with an inverted microscope and the percentage growth recorded. If confluence was <60–80%, a change of medium was performed until cells covered 80% surface of culture. Then, dissociation and cellular expansion (passage) were carried out and the subcultures developed in order to increase and purify the MSC cell line. The cells obtained during this first step were cryopreserved in FBS and 10% DMSO (Dimethyl sulfoxide, Sigma Ref. D2650), and stored in liquid nitrogen at −196°C. Then, when the cells were needed for the assays, they were thawed at 37°C, seeded at a density of 1000 cells/cm^2^ and kept in culture for ∼7–10 days before use, changing the medium every 3 or 4 days.

### ELR biosynthesis and purification

**Figure 1:**
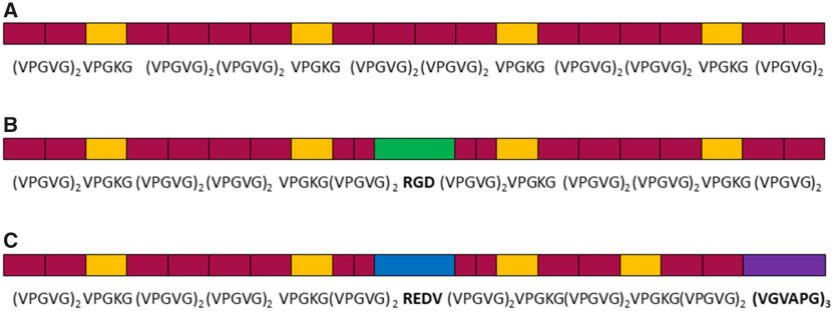
Graphical scheme of the ELR compositions: (**A**) VKVx24; (**B**) HRGD_6_; (**C**) REDV

The gene construction was performed by molecular biology and recombinant DNA technique following standard methods previously described [37, 38]; the purification process was carried out by several centrifugations preceded by inverse transition cycling. The ELRs obtained in this manner were dialyzed against MilliQ water and lyophilized. Three ELRs extensively studied by Gonzalez *et al*., namely VKVx24, HRGD_6_ and REDV, were employed in this study [28] (Fig. 1). HRGD_6_ was designed to contain the extensively studied RGD sequence, which supports cell adhesion via integrins; REDV was designed to contain bioactive sequences such as the CS5 human fibronectin REDV for efficient cell attachment and VGVAPG as an elastase target domain (human leukocyte elastase I). The ELRs were further characterized by electrophoresis gel (SDS-PAGE), mass spectroscopy (MALDI-TOF), nuclear magnetic resonance (NMR), infrared spectroscopy (FTIR) and differential scanning calorimetry (DSC) [39]. The ELRs obtained were chemically modified and characterized by transformation of the -amine group in the lateral lysine chain to produce the cyclooctyne and azide groups necessary for subsequent ‘click chemistry’ reactions, as reported previously [28, 40]. The characterization results are provided in the Supplementary Figs S1–S9.

### Gel formation

Freeze-dried ELRs were dissolved in plain DMEM for 16 h at 4°C at a concentration of 75 mg/ml. The ELR-cyclooctyne solution comprised entirely VKVx24-cyclo, whereas the ELR-azide solution comprised REDV-N_3_ and HRGD_6_-N_3_ (in equal amounts). To prepare the hydrogel embedded with rMSCs, the cells were mixed with the solution of VKVx24-cyclo and dissolved in neat DMEM at 4°C. For gel formation, cold solutions of VKVx24-cyclo and REDV-N_3_ and HRGD_6_-N_3_ were mixed together and the gel formed using catalyst-free click reactions between an azide group and an activated cyclooctyne group.

### Rheological characterization

Rheological experiments were performed using a strain-controlled AR-2000ex rheometer (TA Instruments) with the hydrogel submerged in water. Cylindrical swollen gel samples were placed between parallel, non-porous stainless steel plates (diameter = 12 mm). The gap between the plates was adjusted by applying the minimum normal force to prevent slippage. Before the measurements started, all the samples were relaxed until equilibrium; the temperature was controlled and maintained at 37°C using a Peltier device. Shear deformation measurements were carried out. The dynamic shear modulus was measured by performing a dynamic strain sweep with amplitudes having a range between 0.1 and 20% at a fixed frequency of 1 Hz. Thus, the linear region of viscoelasticity was determined. Afterward, a dynamic frequency sweep was carried out between 0.05 and 70 Hz at a fixed strain amplitude (1%), thus the dependence of the dynamic shear modulus and the loss factor on the frequency was obtained. Finally, the rheological characterization presented the storage modulus and the loss modulus, G′ and G″ respectively. As a results of those, the magnitude of the complex modulus |G*| (|G*|^2^ = (G′)^2^ + (G″)^2^), and the loss factor (tan δ ≡ (G″)/(G′), where δ is a function of frequency or strain amplitude) were calculated.

### Scanning electron microscopy

The morphology of the hydrogel was investigated by scanning electron microscopy (SEM) using a FEI Quanta 200 FEG instrument. No coating procedures were used during the sample preparation; briefly, hydrated hydrogels were submerged into liquid nitrogen, mechanically fractured and freeze-dried. Afterwards, the pictures were collected using the microscope at Landing E of 7.00 keV and a pressure of 0.7 Torr and finally the images were analysed using Image-J software.

### Cell viability assay

The viability of isolated rMSCs embedded in ELRs at 75 mg/ml was evaluated using the Alamar Blue assay (Invitrogen) according to the manufacturer’s guidelines. Briefly, rMSCs were isolated according to the protocol described above and mixed with the hydrogels at a concentration of 8 million cells/ml. A 100-μl aliquot was then pipetted into a 24-well Transwell^®^ tissue culture plate. After allowing the cells to adapt for 4 h, the hydrogels were washed twice with PBS and metabolic activity measurements were conducted at 0, 3, 6, 9, 12 and 15 days of culture. For this purpose, 2 ml of a DMEM-containing 10% Alamar Blue solution was used to replace the culture medium and the cells were incubated in darkness for 2 h at 37°C and under a 5% CO_2_ atmosphere. Subsequently, 70 µl of the reduced medium was transferred to a 96-well plate. The hydrogels were washed twice with PBS and the corresponding growth medium was added and incubated again in order to determine the metabolic activity at different times. Fluorescence (excitation: 560 nm; emission 590 nm) was measured using a SpectraMax M5e (Molecular Devices) microplate reader [41]. The fluorimetric reduction of 10% Alamar Blue reagent in the culture medium by the cells was measured at regular time intervals. Samples for the phase-contrast epifluorescence were fixed at 4% paraformaldehyde (Sigma-Aldrich) for 40 min. Staining was carried out after permeabilization of the sample with 0.2% Triton X-100 (Sigma-Aldrich) and stained with the fluorescent dyes Phalloidin–Alexa Fluor488R and DAPI (Invitrogen).

### *In vivo* experimental model

**Figure 2:**
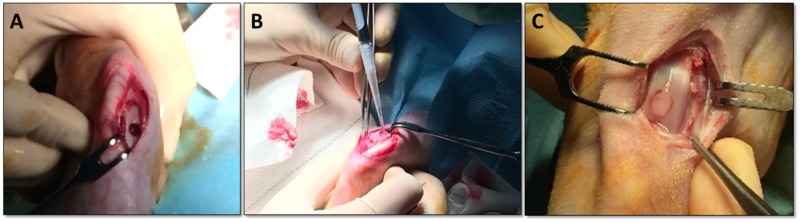
(**A**) Creation of the osteochondral defect with critical size; (**B**) pipetting of the ELR solutions with and without cells embedded inside the defect; (**C**) formation of the gel via a catalyst-free click reaction, thereby entirely filling the lesion created

Ten female New Zealand white rabbits with an age of 6 months and an average weight of 3 kg were used for the creation and treatment of the osteochondral defects. The number of animals was determined by power analysis and consideration of previous studies [42–44], following the 3Rs principles formulated by Russell and Burch for animal experimentation [45]. The animals were anesthetized intramuscularly with medetomidine (0.5 mg/kg) (Braun) and ketamine (25 mg/kg) (Richter Pharma). Afterward, both knees were shaved and cleaned. The surgical procedure involved a parapatellar incision of the skin, which was performed under sterile conditions in order to expose the distal femur. A critical-size (4 × 4 mm full-thickness) osteochondral lesion was created with a drill (Fig. 2A), following well-established surgical procedures [46–49]. The defect was deep enough to reach the osteochondral bone. The ELR-cyclooctyne and ELR-azide solutions were then mixed together and the cold solution (below *T*_t_) was pipetted completely into the defect (Fig. 2B). The gel was immediately formed by a catalyst-free click reactions between the azide group and activated cyclooctyne groups, filling the lesion created with the drill entirely (Fig. 2C). Each animal was surgically operated at both knees and hydrogels with and without rMSCs embedded were pipetted into the right/left knee defects at random. Carprofen (50 mg/kg) (Norbrook) was administrated 4 h after the surgical procedure. All animals were fed and watered *ad libitum* during the study period and maintained in individual cages. Animals were euthanized intravenously with pentobarbital (200 mg/kg) at 4 months post-treatment and the distal femora extracted for further analysis [17].

### Gross morphology

The entire knees of each rabbit were dissected and the distal part of each femur was extirpated. Samples for each group were photographed and examined for evaluation as per the International Cartilage Repair Society (ICRS) gross morphology assessment scale for cartilage repair [50, 51].

### Histological analysis

A blind macro- and microscopic analysis was performed by trained histologists for all the samples previously fixed in 4% formaldehyde in PBS 0.05 M (pH 7.3) at 4°C. The sections were stained with several stains: Hematoxylin and Eosin (H&E), Picro-Sirius Red Stain and Safranin-O/Fast Green, for collagen and glycosaminoglycan (GAGs) stains, respectively. The staining procedures were performed according to common methods. Moreover, immunohistochemistry was performed with primary antibody Mouse monoclonal anti-collagen type I and anti-collagen type II. Samples from each rabbit (*n* = 10 for each group) were graded by two observers using the ICRS visual histological assessment scale for cartilage repair [52].

### Statistical analysis

Values are expressed as mean±standard deviation. Statistical analysis was evaluated by one-way analysis of variance using the Tukey’s method. *P-*values <0.05 was considered statistically significant.

## Results

### Rheological characterization

**Figure 3:**
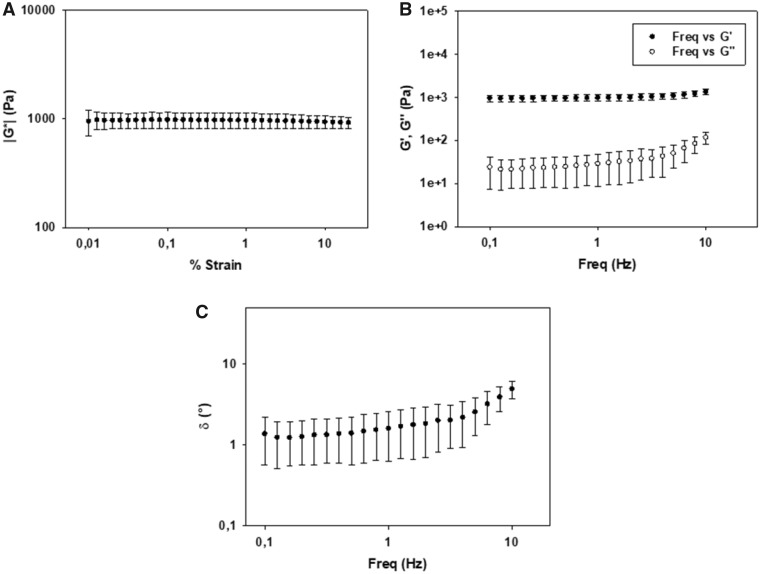
Rheological measurement for the ELR hydrogel at 37°C and 75 mg/ml. (**A**) Strain dependence of the complex modulus (|G*|); (**B**) frequency dependence of the storage (G′) and loss (G″) modulus; (**C**) frequency dependence of δ. Each curve corresponds to the average of three different sample measurements

The linear viscoelastic region of the ELR hydrogels comprising 50% VKVx24-cyclo, 25% REDV-N_3_ and 25% HRGD_6_-N_3_ at 75 mg/ml was determined by using strain sweep measurements from 0.01 to 20% strain at a frequency of 1 Hz (Fig. 3A). The complex modulus (|G*|) at 75 mg/ml shows a constant value of 964 ± 156 Pa (at 1% strain) in this strain range. As such, a 1% strain was selected to carry out the dynamic frequency sweep measurements. Evolution of the storage (G′) and loss moduli (G″) is represented in Fig. 3B. At a frequency of 1 Hz, the value of G′ is 960 ± 162 Pa, whereas the value of G″ is 28 ± 19 Pa. Moreover, the evolution of δ as a function of the frequency is represented in Fig. 3C (the value of δ at 1 Hz is 1.6 ± 0.9°).

### SEM

**Figure 4:**
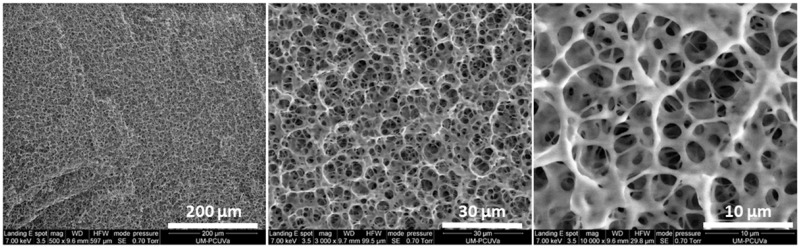
Representative SEM images for the ELR hydrogel at 75 mg/ml and different magnifications

ELR hydrogels at 75 mg/ml show a porous environment, with pore sizes ranging from around 3 to 20 µm and a wall thickness of 1.11 ± 0.34 µm (Fig. 4). This large variety of pore size is due to the internal interconnected structure of the ELR, where small pores are able to merge to form larger pore structures.

### Cell viability assay

**Figure 5:**
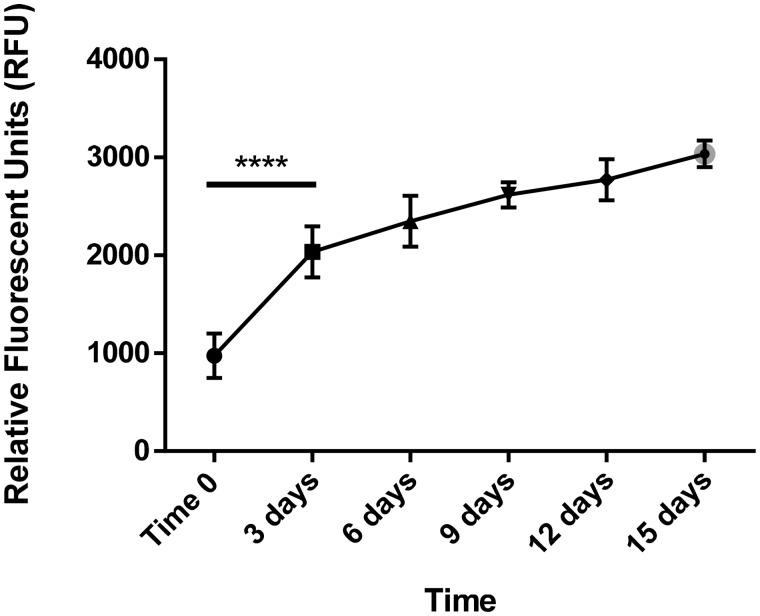
Cell viability test of a 3D ELR gel (75 mg/ml) embedded with rMSCs at different time points (****P < 0.0001)

A cell viability assay was performed for 2-week culturing of the ELR hydrogel at 75 mg/ml when embedded with rMSCs (8 million/ml). Assay data were recorded at different time points (0, 3, 6, 9, 12 and 15 days) in order to gain a better understanding of the metabolic activity of the rMSCs. The cell viability analysis revealed an increment in metabolic activity, with a significant difference between 0 and 3 days and a constant increase from day 3 to 15 during the culture process (Fig. 5). The biocompatibility demonstrated by our ELR-based hydrogel is in agreement with similarly cross-linked hydrogels previously studied [53]. Moreover, the curve trend of this viability assay was in accordance with typical cell-growth behavior, whereby the number of cells increases exponentially in the first part of the culture, subsequently reaching a stable value.

**Figure 6:**
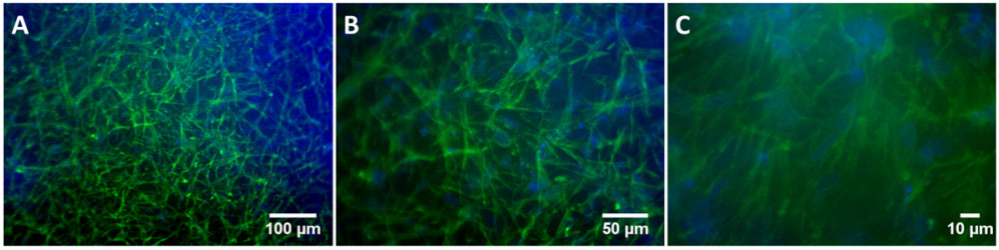
Optical microscope images of hydrogel colonization by rMSCs after culture for 15 days. Pictures collected at different magnifications (**A**–**C**)

Furthermore, the Dapi/Phalloidin analysis (Fig. 6) showed the morphology of the rMSCs embedded in the 3D structure after 15 days of culture. Mesenchymal stem cells are pluripotent cells that are able to differentiate into multiple cell types widely used in both TE and regenerative medicine [41]. An extended and elongated cell shape, with long cytoplasmic processes, can be seen in all the different magnifications, thereby confirming colonization of the hydrogel over 15 days. The cells showed a well-spread morphology, with large extensions of their cytoskeleton actin filaments (green stained). The pictures collected at different magnifications (Fig. 6A–C) help to visualize both the homogeneous distribution of the rMSCs and the colonization of the hydrogel at different focal points.

### *In vivo* study results

#### Macroscopic observation of repaired cartilage

**Figure 7:**
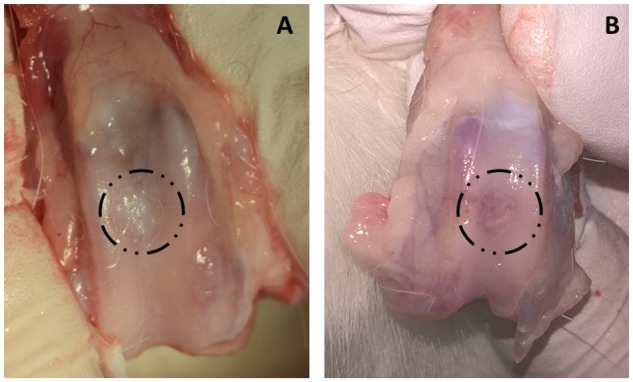
Macroscopic appearance of defects in the trochlear groove (4 mm in diameter) at 4 months post-surgery. (**A**) ELR-based hydrogel embedded with rMSCs; (**B**) ELR-based hydrogel alone. Defects are indicated with a black dashed line

Rabbits were euthanized 4 months after the surgical procedure and the performance of cartilage repair initially evaluated by macroscopic observation. The surface of the defects (Fig. 7) showed that the defects in the central area of the trochlea were completely filled at 4 months post-surgery in all animals from both groups (ELR hydrogels and ELR hydrogels embedded with rMSCs). In addition, the defects were covered by a white layer of fibrous tissue in both groups. The regenerated tissue had a grayish color and could be easily recognized in both cases. As such, the regenerated tissue showed a good integration with the surrounding tissue; indeed, there was no clear boundary between the injured region and the surrounding chondral tissue. The regeneration rate was further evaluated based on macroscopic observation of the regenerated knee cartilage.

**Figure 8:**
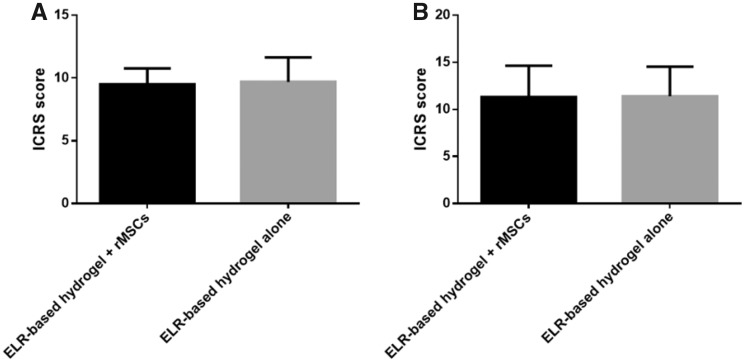
ICRS macroscopic assessment scale. (**A**) Gross morphology assessment; (**B**) histological and immunohistochemical assessment. Values are expressed as mean ± standard deviation (*n* = 10)

As noted above, the samples were evaluated using the ICRS gross morphology assessment scale. Briefly, this gross evaluation takes into consideration three parameters, namely the degree of defect repair, integration with the border zone and macroscopic appearance [54]. Each of these parameters is evaluated on a scale of 0–4, with a total score ranging from 0 to a maximum of 12. The average score for the ELR hydrogel group was 9.7 ± 1.3, whereas the ELR hydrogel embedded with rMSCs scored 9.5 ± 1.9 (Fig. 8A).

### Histological analysis of repaired cartilage

**Figure 9:**
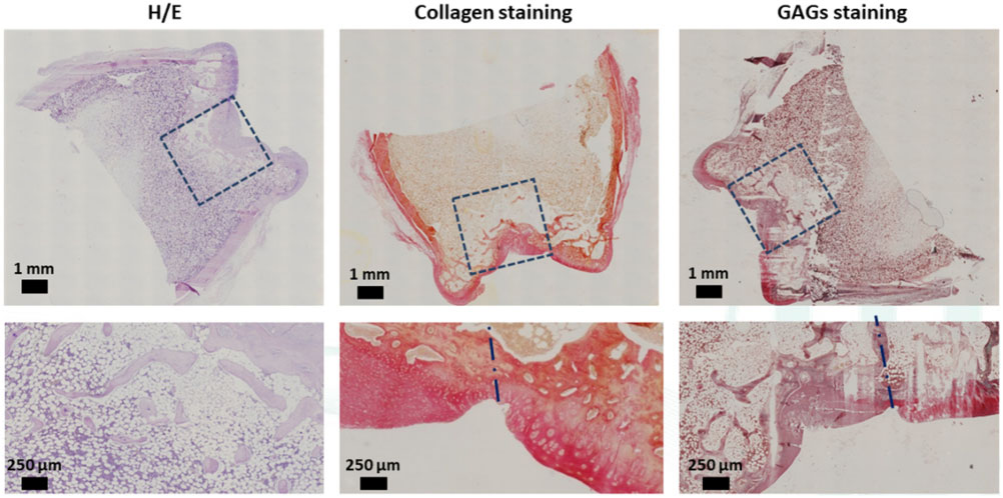
Representative histological staining of repaired cartilage for ELR-based hydrogel with rMSCs

**Figure 10:**
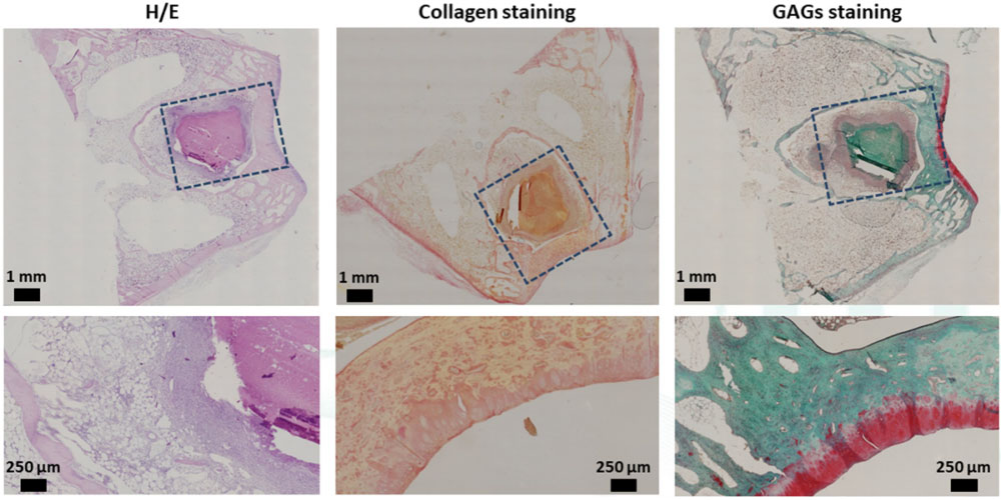
Representative histological staining of repaired cartilage for the ELR-based hydrogel alone

Histological analyses were performed on the sections of the ELR-based hydrogel embedded with rMSCs (Fig. 9) and on the ELR-based hydrogel alone (Fig. 10). For histological analysis, all the sections were stained with H/E, Picro-Sirius Red Stain and Safranin-O/Fast Green, for morphological evaluation and detection of collagen and GAGs, respectively.

Histological analysis of the ELR-based hydrogel embedded with rMSCs (Fig. 9) shows the absence of the hydrogel and that *de novo* bone tissue formation is present. It can also be seen that the new bone tissue exhibits the same porous and morphological structure as the native surrounding tissue. The upper bone region (underneath the cartilage layer) shows a less intense staining due to the degradation of the hydrogel combined to the uncompleted regeneration of the bone layer. Moreover, the collagen staining has the same intensity when comparing the regenerated cartilage with the native one. The regenerated cartilage layer also contained small egg-shaped cells, which is typical of the fibrocartilage-like tissue. Moreover, although GAG staining revealed glycosaminoglycan’s production and secretion in the cartilage layer, metachromatic Safranin-O staining appeared to be less intense for the regenerated cartilage than for the surrounding cartilage. Furthermore, although the regenerated tissue at the articular surface of the samples exhibited an adequate thickness in comparison with the adjacent non-injured articular cartilage, the tissue had a fibrotic appearance. Finally, the subchondral bone was mostly regenerated.

The first aspect that can be seen from the histological analysis of the ELR-based hydrogel alone (Fig. 10) is the continued presence of the hydrogel within the created defect. Although the hydrogel remained intact in the inner part, it started to degrade from the periphery toward the center of the hydrogel. H/E staining clearly showed a difference between native bone tissue and the hydrogel. In addition, in the boundary area of the hydrogel, a higher concentration of cells (revealed by the higher intensity of the staining) enrolled in degradation of the hydrogel and in *de novo* formation of bone tissue can be seen. Safranin-O staining revealed the presence of proteoglycan in the relatively thin repaired tissue. In addition, collagen staining indicated that the new tissue secretes an extracellular matrix. Histological staining revealed a columnar arrangement of the chondrocytes (typical of native cartilage) in the regenerated cartilage. The peripheral migration of these types of cells from the surrounding tissue toward the defect area displayed a smooth and regular surface of the regenerated cartilage, which exhibited a complete integration with the adjacent non-injured cartilage. Moreover, the regenerated cartilage showed no structural differences with respect to healthy cartilage.

The section of the ELR-based hydrogel embedded with rMSCs and the section of the ELR-based hydrogel alone were further analysed by immunohistochemistry with primary antibody anti-collagen type I (fibrocartilage) and anti-collagen type II (hyaline cartilage), for detection of different types of collagen previously revealed by the general Picro-Sirius stain.

**Figure 11:**
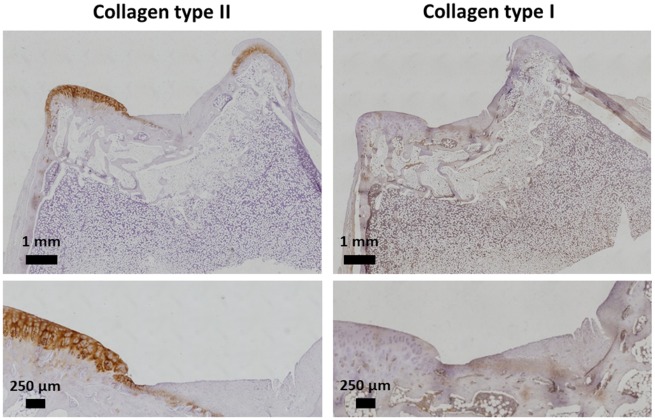
Representative immunohistochemistry study with anti-collagen type I and II for the cartilage regenerated using ELR-based hydrogels with rMSCs

In the case of the ELR-based hydrogel embedded with rMSCs (Fig. 11), no collagen type II was detected in the regenerated cartilage. This result is in accordance with the histological analysis previously described, where a non-columnar arrangement of chondrocytes was revealed. The presence of collagen type II in the native cartilage ensures a correct staining performed for collagen type II. Moreover, the staining for collagen type I appears in a spot-like distribution throughout the section, possibly due to high exposure to this antibody, which is the signal for the non-appearance of collagen type I in the regenerated area. We can, therefore, conclude that the regenerative tissue in the ELR-based hydrogel embedded with rMSCs was mainly fibrous tissue with a small amount of hyaline-like tissue.

**Figure 12:**
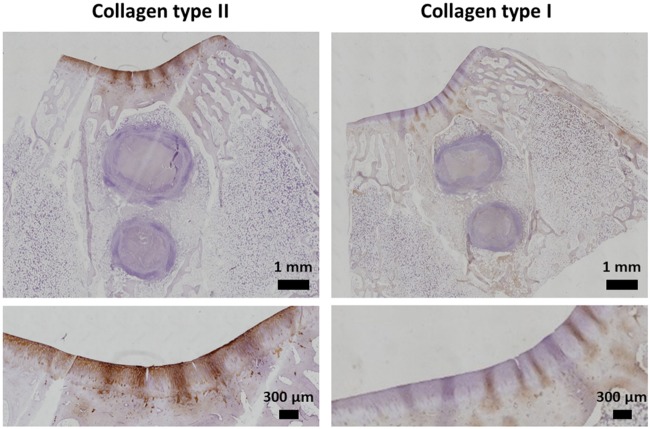
Representative immunohistochemistry study with anti-collagen type I and II for the cartilage regenerated using the ELR-based hydrogel alone

Notably, in the case of the ELR-based hydrogel alone (Fig. 12), a marked production of collagen type II revealed the presence of hyaline cartilage in the regenerated layer. In addition, this result is in accordance with the histological analysis described previously, which exhibited a columnar disposition of the chondrocytes. Moreover, the immunohistochemistry study revealed how the chondrocytes involved in the regeneration process did not produce collagen type I, showing the absence of fibrocartilage.

As reported above, the samples were evaluated according to the ICRS visual histological assessment scale. The resulting score ranges from 0 to a maximum of 18, with the final score being the sum of six parameters, namely surface, matrix, cell distribution, cell population viability, subchondral bone and cartilage mineralization. Each of these parameters is given a value from 0 to 3. The average score in the ELR hydrogel group was 11.4 ± 3.1, whereas the ELR hydrogel embedded with rMSCs scored 11.3 ± 3.3 (Fig. 8B).

## Discussion

It is well known that articular cartilage has a limited regeneration capacity after disease or trauma and that fibrocartilage is produced where the cartilage regeneration takes place [55, 56]. This type of cartilage can easily degenerate and develop into osteoarthritis [57]. Considering that the clinical treatment of defective cartilage remains problematic [58], the purpose of surgery is to regenerate the chondral defects in order to obtain a structurally and biomechanically competent hyaline cartilage. From a functional point of view, clinical treatments are not able to promote the proper regeneration of cartilage defects; TE represents a new approach for articular cartilage repair [51], it consists in reconstructing living tissue by associating cells with biomaterials. The 3D structure of the biomaterial plays a supporting role for the cells, thus helping them to proliferate under physiological conditions [59]. The application of new materials in tissue-engineered scaffolds has received particular interest [60], and several studies have demonstrated how bioinspired materials can simulate the physiological characteristics, thereby enhancing the biological properties of the scaffold [61–63]. In this study, we have designed and developed an ELR-based hydrogel composed by VKVx24-cyclo, REDV-N_3_ and HRGD_6_-N_3_, as reported in Fig. 1. The specific composition of the ELR-based construct has been previously investigated by Staubli *et al*. demonstrating a good composition of the hydrogel tailored for a TE study; indeed, whereas the ELR VKV counterpart gives stability to the hydrogel, the combination of ELRs containing RGD sequence and elastase target domain is crucial for cell infiltration and material colonization [64]. In the light of this previous study, we designed our ELR hydrogel to contain 25% of an ELR bearing the elastase target domain, thus allowing a slower degradation of the scaffold. Moreover, it has to be taken into account that natural polymers showed some limitations in terms of mechanical integrity. Indeed, both collagen and hyaluronic acid have a short lifetime due to degradation by matrix metalloproteinases [65].

The composition of the hydrogel permits immediate gelation by click chemistry as it has been demonstrated by González *et al*. [53], thus conferring the benefit of being an injectable scaffold on our system. The mechanical features of the scaffold are a crucial factor affecting cartilage repair. As it has been demonstrated, chemical cross-linkable ELR hydrogels having similar Molecular Weight to our hydrogel [53, 66] showed no dependence between the swelling ratio and the concentration for the range 50–150 mg/ml at 37°C, maintaining a swelling ratio below 2. On the other hand, the hydrogel’s concentration directly influences the mechanical properties of the hydrogel. Considering the remarkable results obtained in the application of this material at a concentration of 75 mg/ml in TE [28], we decided to use our hydrogel at the same concentration. The rheological characterization of the ELR hydrogel at 75 mg/ml showed a complex modulus of around 1 kPa, which is in accordance with the elastic modulus of many native tissues [67] and with the mechanical features of efficient scaffolds for TE applications [53, 64]. Moreover, the low values of δ obtained for the ELR hydrogel agree with the viscoelastic behavior demonstrated in the cartilage layer [68]. As it has been reported above, the δ is the phase angle between the applied stimulus and the corresponding response as a function of strain amplitude or frequency; the constant values of δ calculated demonstrated a highly elastic energy storing hydrogel at different frequency values. It is important to take into account that articular cartilage has unique biological properties (such as permeability and viscoelasticity) when compared with other cartilage [69]. Indeed, the structure and physiochemical properties of articular cartilage are similar to those of hydrogels. SEM analysis revealed the morphology of the hydrogel at 75 mg/ml, which shows an interconnected structure with adequate porosity and permeability, along with an appropriate pore size for the creation of a 3D scaffold embedded with rMSCs. The pore size determines the exchange of nutrients and waste products because of the void spaces where the cells are seeded and influences *de novo* secretion of ECM [70]. Moreover, the fluid movement in the hydrogel determined by the pore size plays a fundamental role in the regeneration process; in order to guarantee a good regeneration, it should be similar to that for native tissue [71].

Cells play a critical role in the regeneration process; when incorporated into a biomaterial they can enhance tissue regeneration. Although it is well known that chondrocytes only form 1–5% volume of the mature articular cartilage [72], it has been demonstrated that a higher MSCs seeding density results in better chondrogenesis [73–76]. We selected a seeding density of 8 × 10^6^ cells/ml considering the outcomes of previous studies performed with a similar cell density [77, 78]. The cell viability analysis revealed an increment in metabolic activity throughout the 15 days of culture, thus showing that the ELR-based hydrogel is a biocompatible scaffold for cell repopulation. Moreover, considering that the highest increase of metabolic activity was recorded within the first 3 days, the rMSCs appear to be more active when the hydrogel has a lower cell density, reaching a more quiescent state once the hydrogel starts to be repopulated.

The Dapi/Phalloidin analysis showed the morphology of the rMSCs embedded in the 3D structure after 15 days of culture. The specific composition of the ELR hydrogel, which contains RGD and REDV bioactive domains, permitted efficient cell attachment. Indeed, the colonization process indicates that this specific composition of the scaffold is able to support the culture of embedded cells. Assuming that a suitable scaffold for TE should mimic the ECM functional properties, the *in vitro* study showed an adequate composition of the ELR hydrogel, thereby facilitating the encapsulation of reparative cells into a 3D matrix [79]. Moreover, the elastase target domain (VGVAPG sequence) fosters cell-mediated remodeling of the artificial scaffold. In addition, cell proliferation, and thus colonization of the scaffold, is guaranteed because of the action of proteases during the synthesis of new extracellular matrix.

In this study, we tested the ELR-based hydrogel embedded with rMSCs and the ELR-based hydrogel alone to repair cartilage defects *in vivo*. Macroscopic examination of the surface of the defects (Fig. 7) showed that the defects were completely covered 4 months after the surgery in all animals. The scores on the ICRS gross morphology assessment scale for the two hydrogels are practically the same, thus suggesting that both groups aid cartilage regeneration, allowing the defects to be filled. However, histological analysis of the dissected knees was necessary to determine which type of cartilage was regenerated, and whether the gel was fully replaced by newly formed tissue.

However, in contrast to the macroscopic evaluation, the histological analysis showed two different responses from the two groups as regards tissue, bone and cartilage. For the bone area, in the case of ELR-based hydrogel alone, a large quantity of intact hydrogel was present, whereas in the rMSCs group no intact hydrogel was present. In the boundary area of the ELR-based hydrogel alone, it was observed a higher concentration of cells enrolled in degradation of the hydrogel and in *de novo* formation of bone. This inflammatory cells infiltration in the hydrogel and the consequent degradation of the scaffold was mainly due to the presence of the elastase target domain. This behavior is in accordance with previous studies performed with ELR-based hydrogel containing protease target domains [64]. Moreover, in this case, the degradation came only from the surrounding tissue and the tissue-replacement process was not complete at 4 months post-surgery.

Finally, it is important to take into account that the rejection of engraftment depends essentially by the host immune response, whereby the proportion between inflammation and pro-resolution is the key for successful implantation of the engineered tissue [64, 80]. In contrast, the group treated with the ELR-based hydrogel containing rMSCs showed a much more marked degradation. Indeed, in that case, the degradation occurred both from the surrounding tissue and from the cells embedded in the hydrogel. For the cartilage layer, the histological and immunohistochemistry staining showed how the group treated with the ELR-based hydrogel alone exhibited better cartilage regeneration compared to the group treated with the ELR-based hydrogel containing rMSCs. The group with no rMSCs exhibited all the typical features of hyaline cartilage, such as the columnar disposition of chondrocytes, excellent GAG staining and the presence of collagen type II, that provides the tensile ability to the cartilage layer [8]. Moreover, the absence of fibrocartilage confirms that ELR hydrogel is an attractive solution for cartilage regeneration. Although there is no significant difference in the scores for the ICRS visual histological assessment scale, we can conclude that the ELR-based hydrogel containing rMSCs leads to faster regeneration of the bone tissue and worse cartilage regeneration. In contrast, the ELR-based hydrogel alone enhanced the quality of the regenerated cartilage but the degradation of the hydrogel in the bone area was not complete. During the repair process, the hydrogel was gradually replaced by *de novo* tissue formation. Starting with the assumption that the purpose of this scaffold is to promote the bone and cartilage formation instead than merely substitute the tissue, it is important to evaluate either the capacity to support the cell adhesion and proliferation and the mechanical stability at the defect site. A crucial aspect was played by the 25% of the ELR containing the elastase domain, which allows for degradation of the hydrogel. As such, it could be of interest to test other hydrogel candidates that have different percentages of protease sequences in order to synchronize bone regeneration and cartilage repair. Moreover, another important aspect to take into consideration is the proportion between the RGD sequence and the REDV domain; as discussed recently by Flora *et al*., the ratio between these two bioactive domains can tailor the selectivity of the biomaterial toward specific cell lines [81]. Generally speaking, the degradation time of materials should match the production speed of the new tissue. Rapid degradation of the scaffold affects both repopulation of the hydrogel by rMSCs and their differentiation or the colonization of chondrocytes from the surrounding native tissue. A slow degradation could hinder cell proliferation and matrix secretion [82, 83], although we found that, a high density of rMSCs in our scaffold increased the regeneration of fibrocartilage instead of hyaline cartilage. Another important aspect that has to be taken in consideration is the cell–cell contact in the hydrogel, which regulates not only the cell behavior and the MSCs differentiation, but it is also crucial for the development of the tissue architecture. This parameter is strongly correlated to the cells density of the hydrogel. One of the major challenges for osteochondral repair is to obtain regenerated cartilage with adequate mechanical properties. This outcome is not completely fulfilled by synthetic hydrogels, which do not show the biological features of ECM and tend to regenerate fibrocartilage [84].

Our ELR hydrogel has been shown to have an adequate composition, with tunable degradation rate and adhesion behavior, exhibiting a good balance between the degradation rate and adhesion behavior, and allowing for the colonization of chondrocytes with an optimal secretion of extracellular matrix-collagen type II at the periphery of the hydrogel. Moreover, we observed excellent cartilage repair without the need for cellular implantation, which is a significant advantage in terms of eluding all the technical and ethical complications of cell implantation. Finally, these results are promising as regards the testing of other ELR-based hydrogels with higher degradation rates for bone regeneration, thus leading to an optimal system for osteochondral repair.

## Conclusion

One of the biggest challenges in TE is to discover a new biomaterial that guarantees an adequate regeneration of either bone or cartilage tissue. In this study, we took advantage of the recombinant DNA technique to develop a bioactive ELR-based hydrogel with a specific composition as an injectable scaffold for osteochondral repair. The specific composition of this hydrogel allowed for faster bone regeneration when embedded with rMSCs compared to the injection of the hydrogel alone. Similarly, the specific composition of this bioactive hydrogel allowed for the infiltration and the recruiting of native cells (chondrocytes) to promote the repair and remodeling of articular cartilage. According to the outcomes revealed by this study, a promising therapy for osteochondral repair could be the development of a bilayer system based on ELR hydrogels. This system would consist of a bottom layer composed by the hydrogel embedded with MSCs, which fill the subchondral bone cavity, whereas the upper layer would be composed by the hydrogel itself. In conclusion, our bioactive ELR-based hydrogel alone was able to resemble native tissue in terms of hyaline cartilage content and the absence of fibrocartilage, thus proving to be a promising scaffold for cartilage repair.

## Supplementary Material

rbz023_Supporting_Information
