# Supplementary material for: An elastin-like recombinamer-based bioactive hydrogel embedded with mesenchymal stromal cells as an injectable scaffold for osteochondral repair
Source: Regen Biomater. 2019 May 20;6(6):335–47. doi: 10.1093/rb/rbz023 (PMC6897338; doi:10.1093/rb/rbz023)
Supplement: rbz023_Supporting_Information [file rbz023_supporting_information.docx]

**Supporting Information**

**An elastin-like recombinamer-based bioactive hydrogel embedded with mesenchymal stromal cells as an injectable scaffold for osteochondral repair**

**Filippo Cipriani ^1*^, Blanca Ariño Palao ^2^, Israel Gonzalez de Torre ^1,3^, Aurelio Vega Castrillo ^2^, Matilde Alonso ^1,3^, Angel J Àlvarez Barcia ^4^, Ana Sanchez ^5^, Verónica García ^5^, Monica Lopez Peña ^6^, José Carlos Rodriguez-Cabello ^1,3^**

1: Technical Proteins Nanobiotechnology S.L., Paseo Belén 9A, 47011 Valladolid, Spain

2: Hospital Clínico de Valladolid. Departamento de traumatología, Valladolid, Spain

3: Bioforge, University of Valladolid CIBER-BNN, Paseo de Belén 19, 47011 Valladolid, Spain

4: SIBA – UVa: servicio investigación y bienestar animal, University of Valladolid, Spain

5: Instituto de Biología y Genética Molecular (IBGM), Universidad de Valladolid y CSIC, Valladolid, Spain

6: Facultad de veterinaria, Campus Universitario, Lugo, Spain

**Differential Scanning Calorimetry (DSC) measurements**

DSC experiments were performed using the Mettler Toledo 822^e^ DSC with a liquid nitrogen cooler accessory and under a nitrogen atmosphere. All samples were equilibrated for 5 min at 0 °C inside the sample chamber before the experiment started, and then, heated from 0 to 60 °C with a speed of 5 °C/min.

**Figure S1.** DSC graph of HRGD_6_-N_3_ showing the experimental T_t_ in PBS at physiological pH.

**Figure S2.** DSC graph of REDV-N_3_ showing the experimental T_t_ in PBS at physiological pH.

**Figure S3.** DSC graph of VKV-Cyclo showing the experimental T_t_ in PBS at physiological pH.

**Fourier Transform Infrared Spectroscopy (FTIR).**

FTIR analysis was carried out using a Bruker FTIR spectrophotometer (Bruker, USA), whereas the spectral calculations were performed by the OPUS (version 4.2) software (Mattson Instrument INC.). For each spectrum, a 512-scan interferogram was collected at single beam absorption mode with a 2 cm^-1^ resolution within the 4000- 600 cm^-1^ region. Measurements were performed in triplicates and averaged to obtain the final FTIR absorption spectrum of the sample. Residual water vapour absorption was interactively subtracted from the sample spectra.


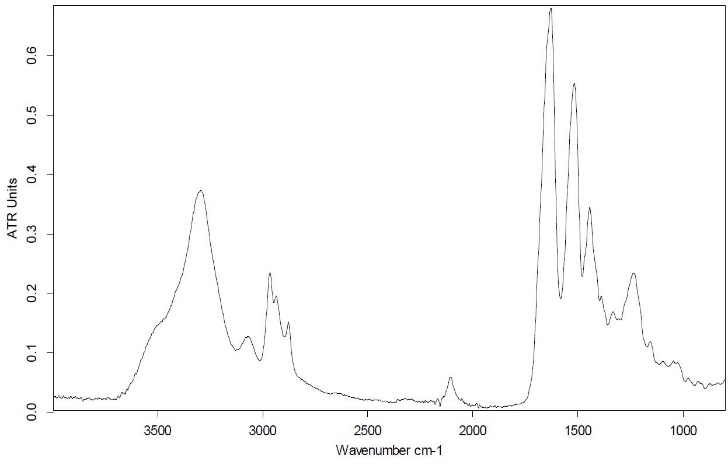


**Figure S4.** FTIR of HRGD_6_-N_3._

**Figure S5.** FTIR of REDV-N_3._

**
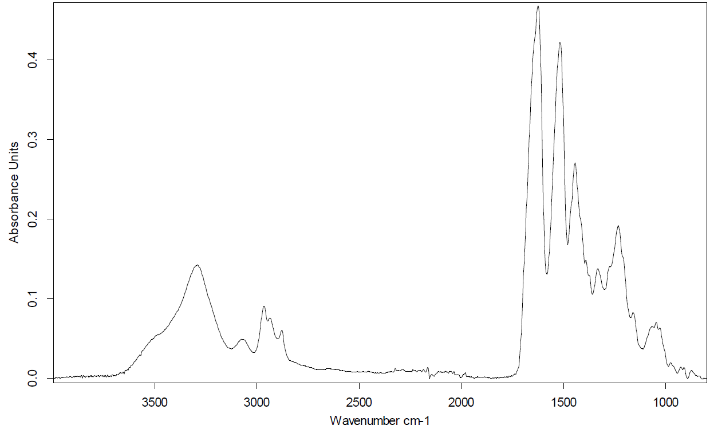
**

**Figure S6.** FTIR of VKV-Cyclo.

**Proton nuclear magnetic resonance ^1^H-NMR Spectroscopy**

NMR analysis was carried out using a 400 MHz Agilent Technologies equip with an Agilent MR console 400 and a One NMR probe. The measurements were carried out at 298 K with samples of 20–30 mg of the modified elastin like recombinamers, purified, and dissolved in DMSO-d6. Chemical shifts (δ) are given in ppm.

The no deuterated dimethyl sulfoxide peaks at d ¼ 2.5 ppm and d ¼ 39.51 ppm were used as internal reference for 1H and 13C NMR spectra, respectively.


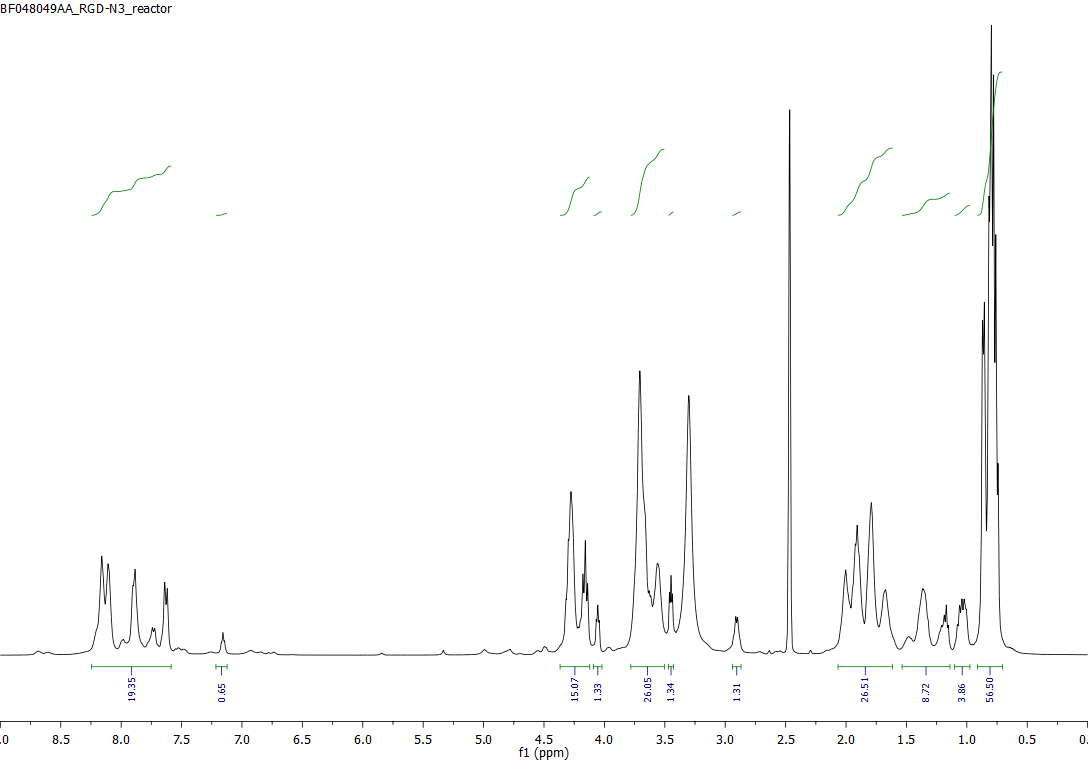


**Figure S7.** H-NMR spectrum of HRGD_6_-N_3_ showing the integration of the peaks corresponding to the different types of hydrogens.


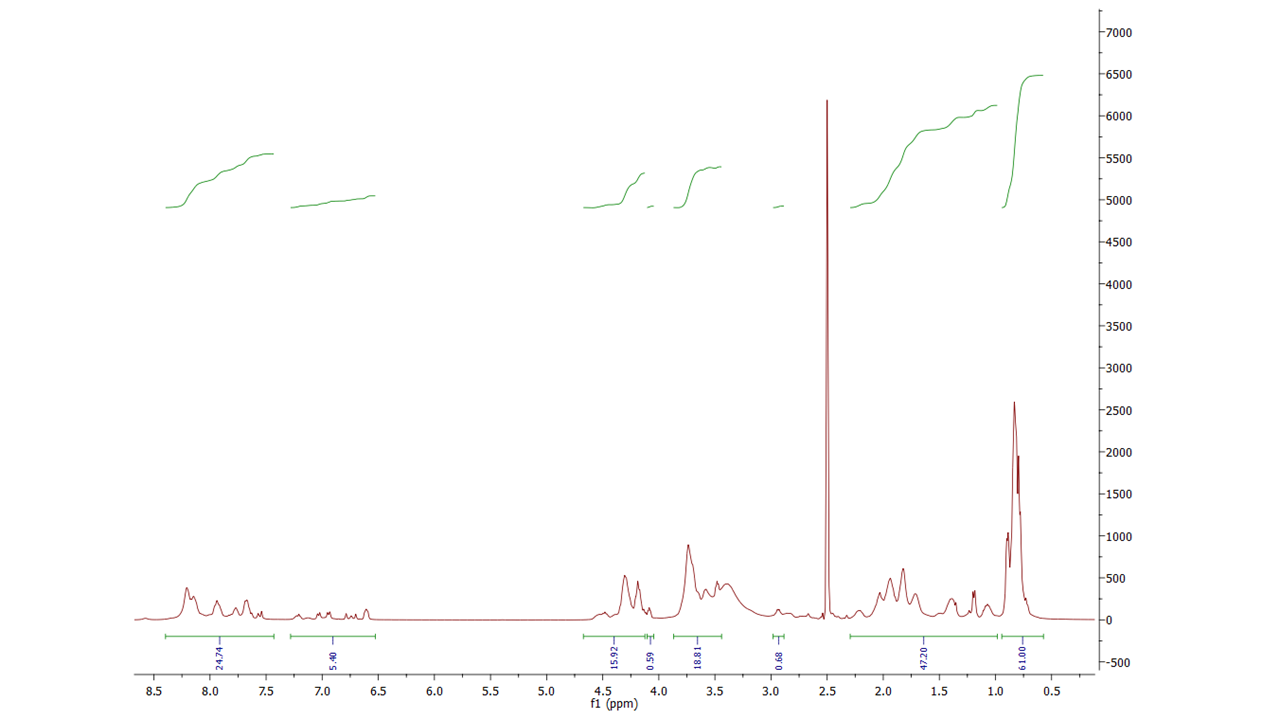


**Figure S8.** H-NMR spectrum of REDV-N_3_ showing the integration of the peaks corresponding to the different types of hydrogens.


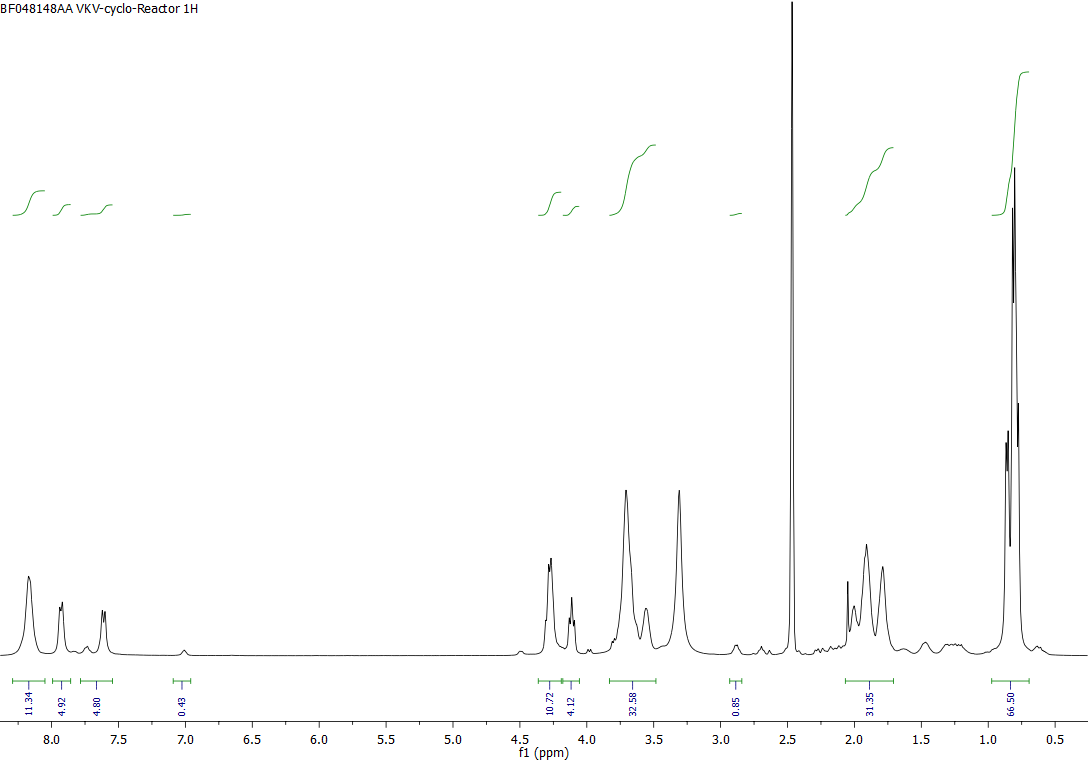


**Figure S9.** H-NMR spectrum of VKV-Cyclo showing the integration of the peaks corresponding to the different types of hydrogens.
